# Statins change the cytokine profile in *Trypanosoma cruzi*-infected U937 macrophages and murine cardiac tissue through Rho-associated kinases inhibition

**DOI:** 10.3389/fimmu.2022.1035589

**Published:** 2023-01-11

**Authors:** Fabiola González-Herrera, Natasha S. Clayton, Daniela Guzmán-Rivera, Ileana Carrillo, Christian Castillo, Mabel Catalán, Renatto Anfossi, Helena Quintero-Pertuz, María Elena Quilaqueo, Claudio Olea-Azar, Mario Rivera-Meza, Ulrike Kemmerling, Anne J. Ridley, Raúl Vivar, Juan Diego Maya

**Affiliations:** ^1^ Molecular and Clinical Pharmacology Program, Instituto de Ciencias Biomédicas, Faculty of Medicine, University of Chile, Santiago, Chile; ^2^ School of Cellular and Molecular Medicine, Faculty of Life Sciences, University of Bristol, Bristol, United Kingdom; ^3^ Escuela de Farmacia, Facultad de Medicina, Universidad Andrés Bello, Santiago, Chile; ^4^ Núcleo de Investigación Aplicada en Ciencias Veterinarias y Agronómicas, Facultad de Medicina Veterinaria y Agronomía, Universidad de Las Américas, Santiago, Chile; ^5^ Department of Chemical Pharmacology and Toxicology, Faculty of Chemical and Pharmaceutical Sciences, University of Chile, Santiago, Chile; ^6^ Department of Inorganic and Analytical Chemistry, Faculty of Chemical and Pharmaceutical Sciences, University of Chile, Santiago, Chile; ^7^ Integrative Biology Program, Instituto de Ciencias Biomédicas, Faculty of Medicine, University of Chile, Santiago, Chile

**Keywords:** chronic chagas cardiomyopathy, statins, rho-kinase, cytokine profile, macrophage polarization, *Trypanosoma cruzi*

## Abstract

**Introduction:**

Chronic Chagasic cardiomyopathy (CCC), caused by the protozoan Trypanosoma cruzi, is the most severe manifestation of Chagas disease.CCC is characterized by cardiac inflammation and fibrosis caused by a persistent inflammatory response. Following infection, macrophages secrete inflammatory mediators such as IL-1β, IL-6, and TNF-α to control parasitemia. Although this response contains parasite infection, it causes damage to the heart tissue. Thus, the use of immunomodulators is a rational alternative to CCC. Rho-associated kinase (ROCK) 1 and 2 are RhoA-activated serine/threonine kinases that regulate the actomyosin cytoskeleton. Both ROCKs have been implicated in the polarization of macrophages towards an M1 (pro-inflammatory) phenotype. Statins are FDA-approved lipid-lowering drugs that reduce RhoA signaling by inhibiting geranylgeranyl pyrophosphate (GGPP) synthesis. This work aims to identify the effect of statins on U937 macrophage polarization and cardiac tissue inflammation and its relationship with ROCK activity during T. cruzi infection.

**Methods:**

PMA-induced, wild-type, GFP-, CA-ROCK1- and CA-ROCK2-expressing U937 macrophages were incubated with atorvastatin, or the inhibitors Y-27632, JSH-23, TAK-242, or C3 exoenzyme incubated with or without T. cruzi trypomastigotes for 30 min to evaluate the activity of ROCK and the M1 and M2 cytokine expression and secretion profiling. Also, ROCK activity was determined in T. cruzi-infected, BALB/c mice hearts.

**Results:**

In this study, we demonstrate for the first time in macrophages that incubation with T. cruzi leads to ROCK activation via the TLR4 pathway, which triggers NF-κB activation. Inhibition of ROCKs by Y-27632 prevents NF-κB activation and the expression and secretion of M1 markers, as does treatment with atorvastatin. Furthermore, we show that the effect of atorvastatin on the NF-kB pathway and cytokine secretion is mediated by ROCK. Finally, statin treatment decreased ROCK activation and expression, and the pro-inflammatory cytokine production, promoting anti-inflammatory cytokine expression in chronic chagasic mice hearts.

**Conclusion:**

These results suggest that the statin modulation of the inflammatory response due to ROCK inhibition is a potential pharmacological strategy to prevent cardiac inflammation in CCC.

## 1 Introduction

*Trypanosoma cruzi* (*T. cruzi*), a hemoflagellate protozoan parasite, is the etiologic agent of Chagas disease (CD), afflicting about six million people in Latin America, where it is endemic, and in other areas due to migration. This disease is characterized by progressing from an acute, often asymptomatic, to a chronic phase, where chronic Chagas cardiomyopathy (CCC) is the most severe presentation (1). The primary determinant of CCC pathogenesis is parasite persistence which induces immune-mediated cardiac damage caused by inflammation and fibrosis. *T. cruzi*-induced heart injury provokes arrhythmias, heart failure, and sudden death (2). The current treatment for CD is benznidazole and nifurtimox, where available. Although these drugs are effective in the acute phase, they do not prevent cardiac events when administered during the chronic phase (3).

Macrophages are the main immune cell target during *T. cruzi* infection, both in the acute and chronic phases, as they bridge the innate and adaptive immune responses (4). Thus, macrophage activation and infiltration are crucial steps in CD (5). These are heterogeneous cells that polarize into classically (M1) or activated (M2) macrophages (6). M1 macrophages secrete pro-inflammatory cytokines such as IL-1β, IL-6, TNF-α, and IFN-γ that contribute to microbial killing. In contrast, M2 macrophages produce regulatory cytokines like IL-10, IL-4, and TGF-β to assist with cell debris clearance and tissue repair (6, 7). Although pro-inflammatory cytokines play a significant role in controlling parasite replication and dissemination in the acute phase (8), their sustained release during the chronic phase contributes to the development of myocardial damage and the onset of symptomatic disease (8, 9). In addition, there are higher M1 and lower M2 serum cytokines in CCC patients than in patients with indeterminate (asymptomatic) chronic CD (10). Moreover, drug-induced polarization towards M2 may reduce inflammation and fibrosis in CCC (11). Therefore, immunomodulatory therapies are a promising strategy for treating CCC.

Ras Homologue (Rho)-associated coiled-coil containing protein kinases (ROCKs) are serine/threonine kinases that control the assembly of the actin filaments and actomyosin contractility and thereby regulate a variety of physiological processes including cell adhesion, migration, phagocytosis, apoptosis, and mitosis (12). The small GTPase RhoA activates the two ROCKs, ROCK1, and ROCK2. The ROCK enzymes participate in leukocyte infiltration in inflammation models such as ischemic heart injury (13, 14) and atherosclerosis (15, 16). There has been increasing evidence suggesting a role of ROCKs in macrophage polarization (17, 18), and as a potential therapeutic target in cardiovascular diseases (19) and in parasitic infections like malaria (20) but their roles have not been investigated in CD.

Statins are 3-hydroxy-3-methylglutaryl coenzyme A (HMG CoA) reductase inhibitors, widely used to treat dyslipidemia. HMG-CoA reductase is the rate-limiting enzyme for mevalonate synthesis and the subsequent formation of cholesterol and isoprenoid intermediates such as farnesyl pyrophosphate (FPP) and geranylgeranyl pyrophosphate (GGPP), required for membrane localization of and signaling by small GTPases, including RhoA (21). Thus, as part of their pleiotropic actions, statins may inhibit ROCK activity in immune cells of atherosclerosis patients (22) and promote M2 macrophage polarization in patients with coronary artery disease (23). We previously demonstrated the effect of statins in decreasing cardiac tissue inflammation and fibrosis in mouse models of chronic Chagas cardiomyopathy (24, 25). However, the impact of *T. cruzi* infection on macrophage polarization and its relationship with ROCK activity has not been studied yet. Thus, in the present study, we evaluated the effect of the statin atorvastatin on *T. cruzi*-induced ROCK activation and its effect on M2 polarization in PMA-induced U937 macrophages.

## 2 Materials and methods

### 2.1 Cell cultures

The human pro-monocytic myeloid leukemia cell line U937 (ATCC^®^ CRL-1593.2, USA) was maintained in RPMI containing 10% FCS, penicillin (100 U/mL), and streptomycin (100 μg/mL) (Thermo Fisher Scientific, USA). For each experiment, U937 cells were differentiated into macrophages by incubation with 10 ng/mL PMA (Sigma-Aldrich, USA) for 48 h; then, the supernatant was removed, and fresh medium was added. Macrophage differentiation was assessed by RT-qPCR of CD14 (**Supplementary Figure 1**). After 48 h in fresh medium, U937 cells were treated for each experiment. *T. cruzi* incubation was always at a multiplicity of infection (MOI) of 1. This ratio was selected because it assures a 1:1 cell contact without jeopardizing cell viability (**Supplementary Figure 2**). Vero cells (ATCC^®^ CCL-81, USA) were cultured in RPMI containing 5% FCS, penicillin (100 U/mL), and streptomycin (100 μg/mL). Vero cells were infected with *T. cruzi* trypomastigotes (Dm28 strain) at an MOI of 5 for parasite amplification. The HEK293T (ATCC^®^ CRL-3216, USA) cell line was cultured in DMEM containing 10% FCS, penicillin (100 U/mL) and streptomycin (100 μg/mL). HEK293T cells were cultured for lentivirus production. All cells were grown at 37°C in 5% CO_2_ in a humidified atmosphere.

### 2.2 Drug and inhibitor treatment

Depending on the experiment, PMA-induced U937 cells were treated with drugs and inhibitors before incubation with *T. cruzi* trypomastigotes. 0.1 to 10 µM Atorvastatin (Sigma-Aldrich, USA) was incubated for 24 h, 10 µM of the ROCK inhibitor Y-27632 (Cell Signaling, USA), 10 µM of the p65 nuclear translocation inhibitor JSH-23 (Sigma-Aldrich, USA), and 1 µM of the TLR4 inhibitor TAK-242 (Sigma-Aldrich, USA) were incubated for 1 h. Dimethyl sulfoxide (DMSO) was used as a solvent at 0.1%. 1 µg/mL of the RhoA inhibitor C3 exoenzyme (26) (Cytoskeleton, USA) was incubated for 24 h, and C3 buffer (500 mM Imidazole, 50 mM Tris HCl, 1 mM MgCl_2_, 200 mM NaCl, 5% sucrose and 1% dextran) was used as a vehicle. 10 µM Geranylgeranyl pyrophosphate ammonium salt (GGPP) (Sigma-Aldrich, USA) was used to study the reversion of atorvastatin inhibition of RhoA prenylation.

### 2.3 Mouse model

The mouse model of CCC used was published previously (25) and authorized by the Bioethics Committee of the Faculty of Medicine, University of Chile (CBA 0937 FMUCH protocol). Briefly, 10^3^ *T. cruzi* trypomastigotes, Dm28 strain, were injected into each experimental mouse intraperitoneally. Sixty days after infection (chronic phase), mice were orally treated with 1 mg/kg/day simvastatin (MSD, United Kingdom) or vehicle (0.5% carboxymethylcellulose) once a day for 20 days. Heart tissue was obtained after euthanasia with a mixture of 100 mg/kg ketamine and 10 mg/kg xylazine on day 80 post-infection for RNA extraction and immunohistochemistry (IHC) analysis.

### 2.4 Western blot

U937 cells were washed with PBS and lysed as described previously (27). Total protein concentration was quantified with Bradford Assay. Extracted proteins were separated by SDS-PAGE (100 V), and transferred to a nitrocellulose membrane (110 V, 90 min). Membranes were blocked in 5% (w/v) non-fat milk powder in TBS-T for 1 h at room temperature (RT), washed with TBS-T, and incubated with primary antibody overnight at 4°C. Membranes were washed and incubated with HRP-conjugated secondary antibodies for 2 h at RT, then washed and incubated with enhanced chemiluminescence (ECL) reagents (Sigma-Aldrich, USA) according to the manufacturer’s instructions. The primary antibodies used were anti-ROCK1 (Cell Signaling, USA #4035), anti-ROCK2 (Cell Signaling, USA #9029), anti-MYPT1 (Cell Signaling, USA #2634), anti-pMYPT1 (T696) (Cell Signaling, USA #5163), anti-p65 (Cell Signaling, USA #8242), anti-p-p65(Ser536) (Cell Signaling, USA #3033), anti-GAPDH (Cell Signaling, USA #2118), and anti-β-actin (Cell Signaling, USA #8457). Secondary antibodies used were anti-mouse-HRP (Santa Cruz Biotechnology, USA SC-516102) and anti-rabbit-HRP (Santa Cruz Biotechnology, USA SC-2357).

### 2.5 Lentivirus generation

Constitutively active ROCK1 and ROCK2, ROCK1 (1-420), and ROCK2 (1-436) have been previously described (28, 29). These proteins were subcloned into the pSEW-GFP plasmid by adding restriction enzyme sites for XhoI and KpnI (NEB) by PCR. The transgene vector (pSEW-GFP, pSEW-GFP-ROCK1(1-420) or pSEW-GFP-ROCK2(1-436)), the gag-pol plasmid (pΔ8.91) and the VSVG-encoding plasmid (pMDM2-G) were transfected in a 4:2:1 proportion into HEK293T cells using 21 μL polyethyleneimine (PEI). Plasmids and PEI were mixed with 500 µL of OptiMEM (Thermo Fisher Scientific, USA) and incubated for 15 min at RT. The mix was added to a T25 flask of 80% confluent HEK293T cells. After 72 h, the supernatants were harvested and filtered through a 45 μm syringe filter to remove cell debris. The filtered supernatant was used immediately or stored at -80°C.

### 2.6 U937 cell transduction

U937 cells (5x10^4^) were resuspended in 500 µL of filtered virus supernatant in a 1.5 mL tube and centrifuged twice for 40 min at 800 x *g*, vortexing between the two centrifugations. Cells and viruses were seeded in a six-well plate and incubated for 48 h. After 48 h, cells were washed and amplified, and 10^6^ cells/mL were sorted for GFP expression by flow cytometry, with a GFP^+^ population > 99%.

### 2.7 RT-qPCR

Total RNA was isolated using TRIzol™ reagent and the PureLink RNA Mini Kit (Thermo Fisher Scientific, USA) according to the manufacturer’s instructions. cDNA was synthesized from 600 ng of total RNA by reverse transcription using M-MLV Reverse transcriptase (Invitrogen, USA) and random primers (Invitrogen, USA). Each reaction mix contained 150 nM of each primer, 1 ng of cDNA, 7 μL of SensiMix^®^ SYBR Green Master Mix (Meridian Bioscience, USA), and H_2_0 for a total of 15 μL. The primers used are listed in **Table 1**.

**Table 1:** Sequences of primers used for the RT-qPCR determinations.

Gene	Species	Fw (5’–3’)	Rv (5’–3’)
*TNF*	Human	TCCCCAGGGACCTCTCTCTA	GAGGGTTTGCTACAACATGGG
*IL6*	Human	CTCAATATTAGAGTCTCAACCCCCA	GAGAAGGCAACTGGACCGAA
*IL1B*	Human	TTCGAGGCACAAGGCACAA	TGGCTGCTTCAGACACTTGAG
*IL10*	Human	CGAGATGCCTTCAGCAGAGT	GGCAACCCAGGTAACCCTTA
*IL4*	Human	CATCTTTGCTGCCTCCAAGAACA	GTTCCTGTCGAGCCGTTTCA
*TGFB1*	Human	CCGTGGAGGGGAAATTGAGG	TGAACCCGTTGATGTCCACTT
*ROCK1*	Human	TGAAAGCCGCACTGATGGAT	GCCATGAGAAAACACATTGCAG
*ROCK2*	Human	TGGGATAACATAAGAGAAACGGC	TGTCATCGAAATTGCTGCTGT
*GAPDH*	Human	AAAGCCTGCCGGTGACTAAC	CCCAATACGACCAAATCAGAGAATA
*ROCK1*	Mouse	CTTTCCTGCAAGCTTTTATCCA	AACGCTCCGAGACACTGTAG
*ROCK2*	Mouse	CTCATCCGAGACCCTCGCTC	ATGCCTTATGACGAACCAACTGA
*IFNG*	Mouse	ACCTGTGGGTTGTTGACCTC	GAGGAACTGGCAAAAGGATGG
*TNF*	Mouse	ATAGCAAATCGGCTGACGGT	CCCTCACACTCACAAACCAC
*IL10*	Mouse	GGGGAGAAATCGATGACAGC	GGTTGCCAAGCCTTATCGGA
*IL4*	Mouse	CTGTGGTGTTCTTCGTTGCTG	CCATATCCACGGATGCGACA
*GAPDH*	Mouse	CTAGGACTGGATAAGCAGGGC	GCCAAATCCGTTCACACCGA
*18S*	*T. cruzi*	GTTCGTCTTGGTGCGGTCTA	TGGAGATTATGGGGCAGT

The amplification was performed in an ABI Prism 7300 sequence detector (Applied Biosystems^®^, USA). The cycling program was as follows: a denaturation step at 95°C for 3 min and 40 amplification cycles of 95°C (15 s), 60°C (15 s), and 72°C (30 s). The final step was a dissociation stage that ranged from 60 to 95°C (100 s). The relative quantification analysis of the results was expressed as an RQ value determined using the comparative control (ddCt) method (30).

### 2.8 Immunofluorescence

U937 cells were seeded in Lab-Tek II Chamber Slides (Thermo Fisher Scientific, USA), and differentiated with PMA as previously described. Cells were treated with inhibitors for 1 h, and then infected with *T. cruzi* for 30 min. After infection, cells were washed and fixed in 4% paraformaldehyde (PFA) for 15 min at RT and blocked with 5% bovine serum albumin in PBS containing 0.1% Triton X-100 for 2 h. Cells were then incubated with monoclonal anti-p65 antibodies (Cell Signaling, USA #8242) overnight at 4°C. The samples were washed with PBS and incubated with Alexa Fluor Ⓡ 555-conjugated anti-rabbit IgG Fab2 (Cell Signaling, USA #4413) for 1 h. Finally, nuclei were stained with DAPI for 5 min, and cells were mounted with Dako Fluorescence Mounting (Dako, USA). The cells were imaged using a Nikon Eclipse 400 epifluorescence microscope (Nikon, Japan), and images were analyzed by mean intensity using ImageJ software (ImageJ 1.47v).

### 2.9 Trypomastigote internalization analysis

U937 cells were seeded in Lab-Tek II Chamber Slides (Thermo Fisher Scientific, USA), and differentiated with PMA as previously described. Cells were dyed with 1 µg/mL Cell Tracker green (Thermo Fisher Scientific, USA) for 30 min, washed, and then incubated with inhibitors. *T. cruzi* trypomastigotes were dyed with 1 µg/mL Cell Tracker orange (Thermo Fisher Scientific, USA) for 30 min, washed, and incubated with the treated U937 with an MOI of 1. After 30 min of parasite incubation, cells were washed, and 24 hours later, cells were fixed with 4% PFA. Fixed cells were dyed with Hoechst (Thermo Fisher Scientific, USA) for 15 min and then mounted with Dako Fluorescence Mounting (Dako, USA). The cells were imaged using a Nikon Eclipse 400 epifluorescence microscope (Nikon, Japan). Parasite internalization was inferior to 5%, without significant differences among the different inhibitors, atorvastatin, and non-treated control (**Supplementary Figure 3**).

### 2.10 Cytokine secretion analysis

U937 cell supernatant was collected and stored at -80°C for cytokine analysis. Samples were analyzed using the MILLIPLEX MAP Human High Sensitivity Panel Immunology Multiplex Assay (Millipore), according to the manufacturer’s instructions. Cytokine levels were read on the Luminex 200 System, Multiplex Bio-Assay (Thermo Fisher Scientific, USA). Cytokine quantification was based on standard curves. Samples for TAK-242 and C3 exoenzyme-treated cells were analyzed with TNF-α and IL-10 Quantikine ELISA kits (R&D Systems, USA). TGF-β levels were measured with the TGF-β1 ELISA kit (Invitrogen, USA). Cytokine levels from ELISA kits were read on the Asys expert plus microplate reader (Biochrom, UK).

### 2.11 Cell viability assay

Cell viability was determined using the methyl-thiazol tetrazolium (MTT) assay. PMA-induced U937 cells were plated in 96-well plates and incubated with atorvastatin or Y-27632, as previously described. After 24 hours, 0.25 mg/ml MTT reagent was added and incubated for 3 h. Precipitates were dissolved in DMSO. The absorption was measured at 570 nm using the Asys expert plus microplate reader (Biochrom, UK).

### 2.12 Cardiac tissue immunohistochemistry

Hearts from the euthanized mice were fixed in 4% formaldehyde (pH 7.3) for 12 h, dehydrated in alcohol, clarified in xylene, and embedded in paraffin to be sectioned at 5 μm. Protein levels were evaluated in cardiac tissues using the immunoperoxidase technique with anti-p-MYPT1 (T696) (Cell Signaling, USA #5163), anti-ROCK1 (Cell Signaling, USA #4035), anti-ROCK2 (Cell Signaling, USA #9029) antibodies. Staining was performed using a peroxidase and diaminobenzidine kit with a chromophore according to the manufacturer’s instructions (RTU-Vectastain kit; Vector Laboratories, USA). The heart tissue was additionally stained with hematoxylin. The images were obtained with a Nikon Eclipse 400 epifluorescence microscope (Nikon, Japan) and analyzed with ImageJ software (ImageJ 1.47v).

### 2.13 Statistical analysis

For all experiments, statistical significance was established at *p*-values of <0.05. The data represent the means and the standard deviations (SD) from at least three independent observations or experiments. All statistical analyzes were performed using GraphPad Prism (9.0) software. One-way or two-way analysis of variance (ANOVA) were performed as appropriate. For a linear correlation, the Pearson correlation coefficient was calculated.

## 3 Results

### 3.1 *T. cruzi* induces ROCK activation through the TLR4 pathway in macrophages

**Figure 1:**
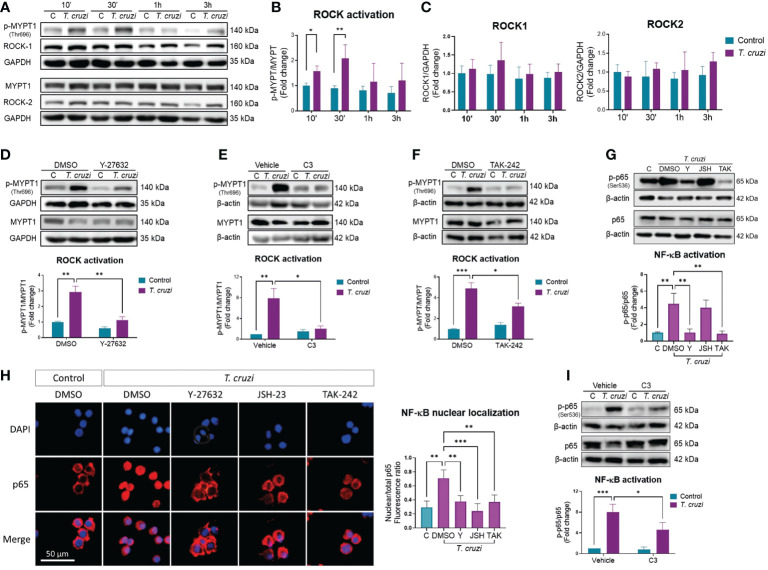
ROCK activation by *T. cruzi* mediates NF-κB activation *via* RhoA/TLR4 in human macrophages. PMA-induced U937 cells were incubated with or without *T. cruzi* trypomastigotes (MOI 1:1) for 10 min, 30 min, 1 h or 3 h. **(A)** Representative western blot. **(B)** ROCK activation was determined as the ratio of phosphorylated (p-MYPT1) to total MYPT1 (MYPT1), relative to control at 10 min. **(C)** ROCK-1 and ROCK-2 protein levels, relative to control at 10 min. **(D)** PMA-induced U937 cells were incubated with the ROCK inhibitor Y-27632 or vehicle (DMSO) for 60 min, and then incubated with or without *T. cruzi* for 30 min. ROCK activation was determined by western blot, as the ratio of p-MYPT1 to total MYPT1. **(E)** PMA-induced U937 cells were incubated with the RhoA inhibitor C3 exoenzyme (C3) or vehicle control (C3 buffer) for 24 h, and then incubated with or without *T. cruzi* for 30 min. ROCK activation was determined by western blot, as the ratio of p-MYPT1 to total MYPT1. **(F)** PMA-induced U937 cells were incubated with the TLR4 inhibitor TAK-242 or vehicle (DMSO) for 60 min, and then incubated with or without *T. cruzi* for 30 min. ROCK activation was determined by western blot, as the ratio of p-MYPT1 to total MYPT1. **(G)** PMA-induced U937 cells were incubated with Y-27632 (Y), JSH-23 (JSH), TAK-242 (TAK) or DMSO (vehicle) for 60 min, and then incubated with or without *T. cruzi* for 30 min. NF-κB activation was determined by western blot as phosphorylated (p-p65) to total p65 ratio. **(H)** Nuclear localization of p65 was determined by epifluorescence microscopy using anti-p65 antibody and DAPI for nuclear stain. P65 nuclear localization was quantified as the ratio between nuclear and total fluorescence. **(I)** PMA-induced U937 cells were incubated with the RhoA inhibitor C3 exoenzyme (C3) or vehicle control (C3 buffer) for 24 h, and then incubated with or without *T. cruzi* for 30 min. NF-κB activation was determined by western blot as p-p65 to total p65 ratio. All data are expressed as mean ± SD of 3 independent experiments. *p<0.05, **p<0.01, ***p<0.001, analyzed by two-way ANOVA and Tukey post-test.

PMA-induced U937 human macrophages were incubated with trypomastigotes at different times to evaluate the effect of *T. cruzi* infection on ROCK activity. ROCK activity was measured as the ratio of phosphorylated to total myosin phosphatase target subunit 1 (MYPT-1) (31). ROCK activation peaked at 30 min (**Figures 1A, B**) without changing ROCK1 or ROCK2 protein levels (**Figure 1C**). Since other kinases, including protein kinase A and protein kinase G, can phosphorylate MYPT-1 T696 (32, 33), PMA-induced U937 cells were treated with the ROCK inhibitor Y-27632 for 1 h and then incubated with *T. cruzi* for 30 min. MYPT-1 T696 phosphorylation was significantly decreased in Y-27632-treated macrophages (**Figure 1D**), confirming that *T. cruzi*-induced MYPT-1 T696 phosphorylation was mediated by ROCK. U937 cells were treated with the RhoA inhibitor C3 exoenzyme to prove the role of RhoA on *T. cruzi*-induced ROCK activation. As expected, RhoA inhibition prevented ROCK activation triggered by *T. cruzi* (**Figure 1E**).

Innate activation upon *T. cruzi* recognition occurs through the Toll-like receptors pathway, including the types 2 and 4 (TLR2, TLR4) (34), which is associated with *T. cruzi*-mediated activation in macrophages and is involved in pro-inflammatory cytokine expression by macrophages (35). Macrophages were pre-incubated with the TLR4 inhibitor TAK-242 for 1 h, and MYPT-1 phosphorylation was determined by western blot analysis to determine whether TLR4 mediates *T. cruzi*-induced ROCK activation. MYPT-1 phosphorylation, and hence ROCK activation, was prevented by TAK-242 (**Figure 1F**), suggesting that TLR4 activation by *T. cruzi* is involved in ROCK activation in macrophages.

### 3.2 ROCK mediates *T. cruzi*-induced NF-κB activation in macrophages

Upon *T. cruzi* recognition by TLR4, the nuclear factor-kappa B (NF-κB) pathways are activated (36). NF-kB is a key transcriptional factor in inducing inflammatory cytokine gene expression (37). Hence, we investigated the role of ROCK on *T. cruzi*-induced NF-κB activation, measuring the phosphorylated to total p65 ratio, and identifying the p65 nuclear localization. *T. cruzi* infection of U937 cells increased p65 phosphorylation (**Figure 1G**) and nuclear localization (**Figure 1H**). Interestingly, ROCK inhibition by Y-27632 prevented *T. cruzi*-induced NF-κB activation. Incubation with JSH-23, a p65 nuclear localization inhibitor (38), did not reduce p65 phosphorylation but prevented p65 nuclear localization, as expected. TLR4 inhibition by TAK-242 prevented p65 phosphorylation and nuclear localization. Additionally, RhoA inhibition with C3 exoenzyme prevented *T. cruzi*-induced NF-κB activation (**Figure 1I**). These data indicate that ROCK may mediate *T. cruzi*-induced NF-kB activation in macrophages, probably through TLR4/RhoA pathway.

### 3.3 ROCK inhibition changes cytokine profile in *T. cruzi*-infected macrophages

**Figure 2:**
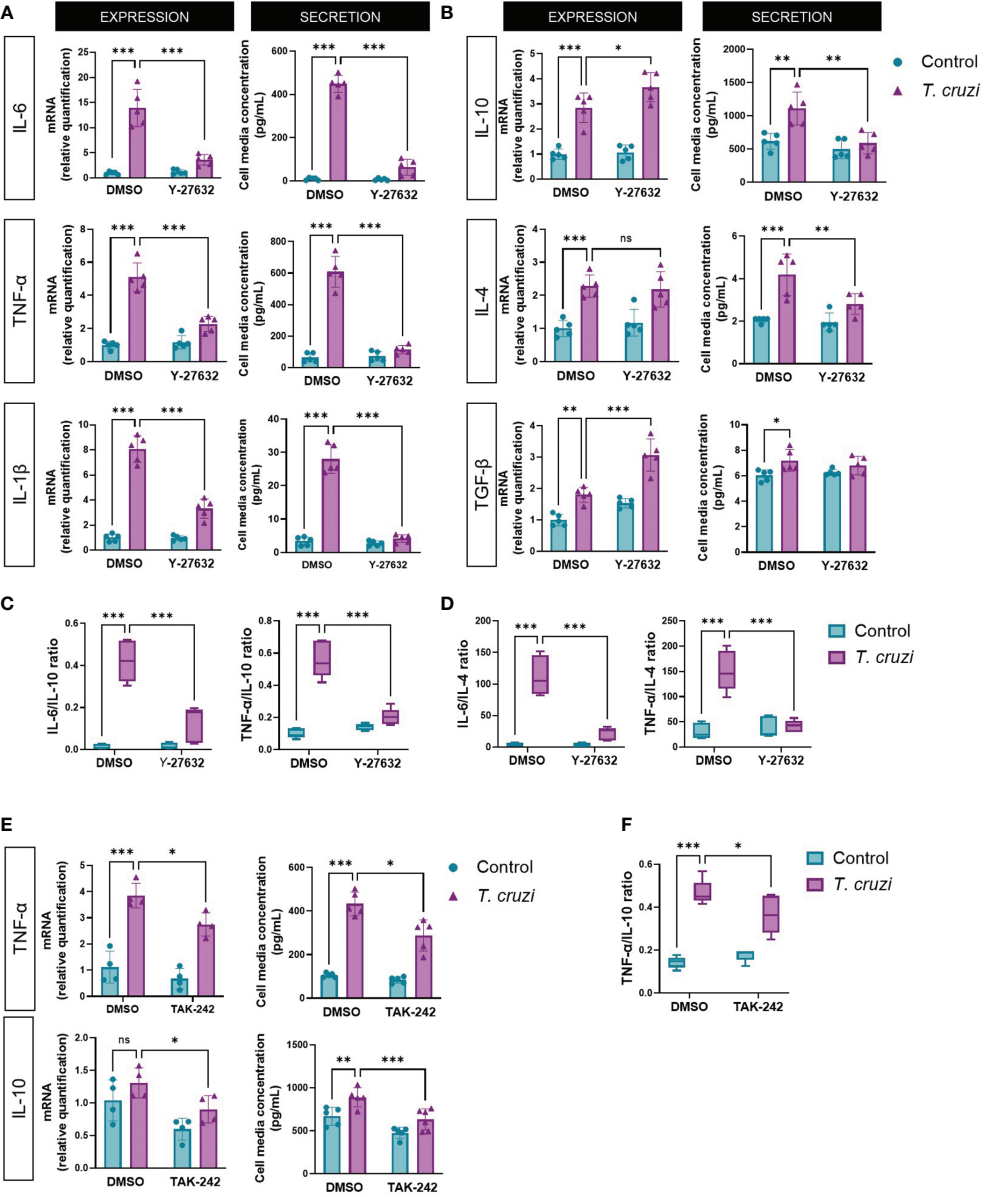
ROCK and TLR4 inhibition decrease M1 phenotype in *T. cruzi*-infected macrophages. PMA-induced U937 cells were incubated with the ROCK inhibitor Y-27632 or DMSO as vehicle control for 60 min and then incubated with or without *T. cruzi* for 30 min. After 6 h post-infection, total RNA was extracted for RT-qPCR analysis, and 24 h post-infection, cell medium was collected for Multiplex analysis of the indicated M1 **(A)** and M2 **(B)** markers. **(C)** Secreted IL-6 and TNF-α to IL-10 ratios. **(D)** Secreted IL-6 and TNF-α to IL-4 ratios. **(E)** PMA-induced U937 cells were incubated with the TLR4 inhibitor TAK-242 or DMSO as vehicle control for 60 min and then incubated with or without *T. cruzi* for 30 min. After 6 h post-infection, total RNA was extracted for RT-qPCR analysis, and 24 h post-infection, cell medium was collected for Multiplex analysis of cytokines. **(F)** Secreted TNF-α to IL-10 ratio. Data are expressed as mean ± SD of 5 independent experiments. *p<0.05, **p<0.01, ***p<0.001, analyzed by two-way ANOVA and Tukey post-test. ns, non significant.

Patients with severe CCC present with a systemic inflammatory profile characterized by a predominant pro-inflammatory cytokine profile that may be attributed to the persistence of an M1 macrophage phenotype due to ROCK activation. Thus, we determined the expression and secretion of M1 (IL-6, IL-1β, and TNF-α) and M2 (IL-10, IL-4, and TGF-β) cytokine markers in *T. cruzi*-infected macrophages. All M1 and M2 markers increased their expression and secretion upon *T. cruzi* infection (**Figure 2**). Interestingly, ROCK inhibition had different effects on M1 versus M2 markers. Y-27632 reduced the expression and secretion of all M1 markers, specifically in *T. cruzi* groups but not in uninfected groups (**Figure 2A**). By contrast, there was a decrease of M2 marker secretion, but relative mRNA levels were unaltered or increased in the ROCK-inhibited cells (**Figure 2B**).

Since cardiac morbidity in chagasic patients is associated with an increased pro/anti-inflammatory cytokine ratio in blood and cardiac tissue (39), we measured IL-6 and TNF-α to IL-10 (**Figure 2C**) and IL-4 (**Figure 2D**) secretion ratios. *T. cruzi* favored a pro-inflammatory balance, while ROCK inhibition favored an anti-inflammatory M2 cytokine profile, similar to the effect observed when RhoA activity was inhibited (**Supplementary Figure 4**). Additionally, we evaluated the effect of TLR4 inhibition on the expression of TNF-α and IL-10, respectively, as representatives of the M1 and M2 phenotypes (**Figure 2E**). Indeed, inhibition of TLR4 activity produced a decrease in the M1/M2 ratio (**Figure 2F**), counteracting the effect produced by TLR4 activation by *T. cruzi*. As ROCK activation occurs at least partially *via* TLR4, this experiment corroborates its role in macrophage polarization.

### 3.4 Atorvastatin inhibits *T. cruzi*-induced ROCK activation

**Figure 3:**
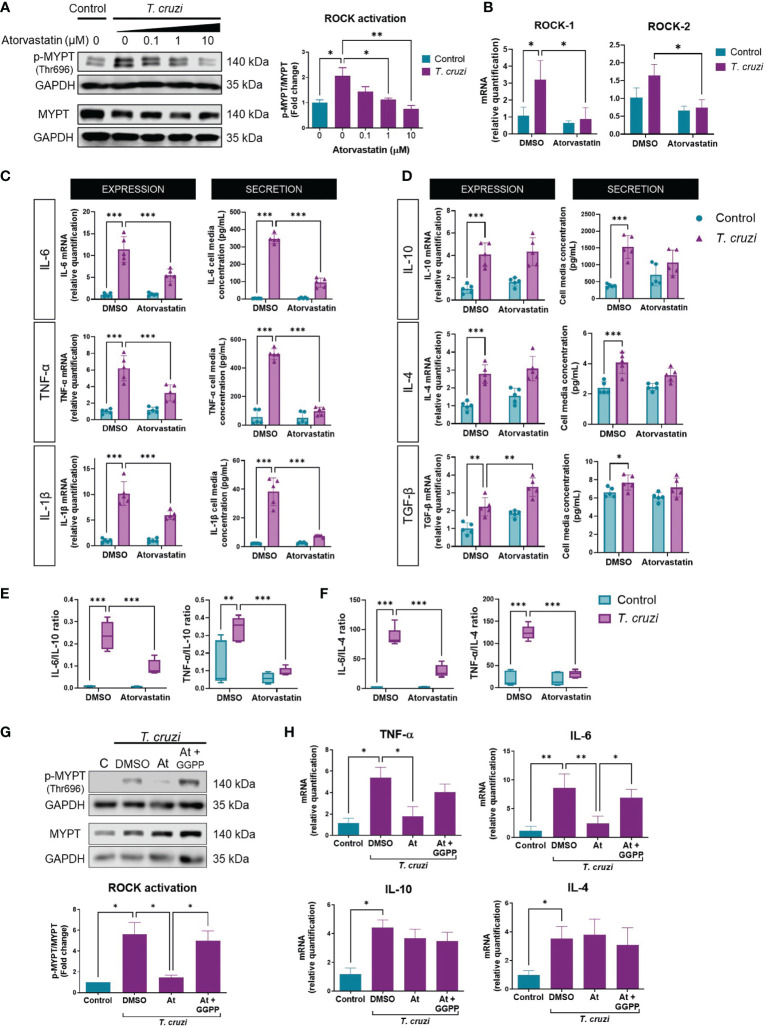
Atorvastatin prevents *T. cruzi*-induced ROCK activation and cytokine profile in macrophages through GGPP depletion. PMA-induced U937 cells were incubated with atorvastatin at increasing concentrations or DMSO (vehicle) for 24 h, and subsequently incubated with or without *T. cruzi* for 30 min. **(A)** Left, representative western blot and right, ROCK activation relative to control, measured as phosphorylated (p-MYPT1) to total MYPT1 ratio. **(B)** 24 h post-infection, total RNA was extracted to determine ROCK1 and ROCK2 mRNA levels relative to DMSO control. **(C)** PMA-induced U937 cells were incubated with atorvastatin or DMSO for 24 h and then incubated with or without *T. cruzi* for 30 min. 6 h post-infection, total RNA was extracted for RT-qPCR analysis, and 24 h post-infection, cell media were collected for Multiplex analysis of M1 and **(D)** M2 markers. **(E)** Secreted IL-6 and TNF-α to IL-10 ratios. **(F)** Secreted IL-6 and TNF-α to IL-4 ratios. **(G)** PMA-induced U937 cells were incubated with atorvastatin or atorvastatin plus 10 µM geranylgeranyl pyrophosphate (GGPP) or DMSO for 24 h, then incubated with or without *T. cruzi* for 30 min. ROCK activation was determined as the ratio of phosphorylated (p-MYPT1) to total MYPT-1 (MYPT1), relative to control, measured by western blot. **(H)** 6 h post-infection, total RNA was extracted for RT-qPCR analysis. Data are expressed as mean ± SD of 3 **(A, B, G, H)** and 5 **(C–F)** independent experiments. *p<0.05, **p<0.01, ***p<0.001, analyzed with two-way ANOVA and Tukey post-test.

As described above, RhoA prenylation is required to activate ROCKs (40); hence, atorvastatin may impede ROCK activation. We, therefore, tested whether atorvastatin altered *T. cruzi*-induced ROCK activation. Atorvastatin treatment decreased *T. cruzi*-induced ROCK activation in a concentration-dependent manner (**Figure 3A**) without altering ROCK1 or ROCK2 protein levels in this time window (**Supplementary Figure 5**). It is necessary to indicate that 10 uM atorvastatin did not alter the viability or morphology of the cells (**Supplementary Figure 2**). Total RNA was extracted 24 h post-infection to determine changes in ROCK expression more extendedly. ROCK1 mRNA increased in *T. cruzi*-incubated macrophages, and atorvastatin prevented this response (**Figure 3B**). ROCK2 mRNA tended to increase in *T. cruzi*-incubated macrophages, but this was not statistically significant (p=0.09). Atorvastatin significantly decreased ROCK2 mRNA expression in *T. cruzi*-infected macrophages. Thus, atorvastatin can prevent *T. cruzi* effects, inhibiting ROCK activity at short times and decreasing ROCK expression at longer times.

Moreover, atorvastatin decreased M1 cytokine mRNA and secretion (**Figure 3C**), and increased M2 cytokine mRNA (**Figure 3D**). Atorvastatin also induced M2 polarization, evaluated as the ratio between M1/IL-10 (**Figure 3E**) and M1/IL-4 secretion (**Figure 3F**). Moreover, adding geranylgeranyl pyrophosphate reversed the rock inhibition (**Figure 3G**) and the anti-inflammatory effect induced by atorvastatin (**Figure 3H**).

### 3.5 Pharmacological ROCK inhibition by atorvastatin changes the phenotype and NF-κB activation of *T. cruzi*-incubated human macrophages

As atorvastatin inhibited ROCK activation and the pro-inflammatory phenotype of *T. cruzi*-infected U937 macrophages, we investigated whether ROCK inhibition induced by atorvastatin is a key mechanism in this effect. To assess this, constitutively active (CA) ROCK1 and ROCK2 were stably expressed in U937 cells by lentiviral transduction (**Supplementary Figure 6**). PMA-stimulated GFP-, CA-ROCK1-, and CA-ROCK2-expressing U937 cells were treated with 10 µM atorvastatin for 24 h, and then infected with *T. cruzi* for 30 min to study macrophage polarization.

**Figure 4:**
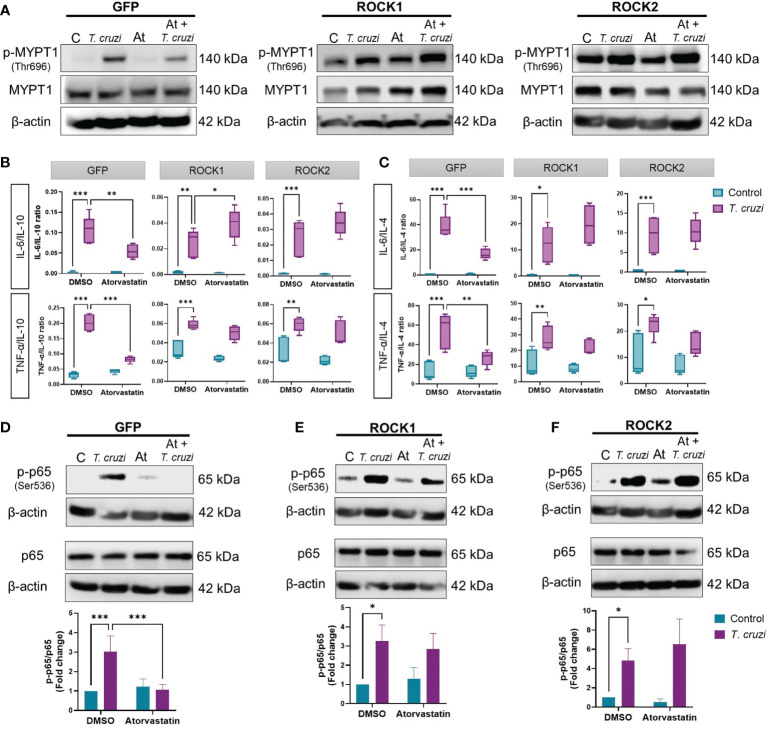
ROCK inhibition is necessary for the anti-inflammatory effect of atorvastatin in *T. cruzi*-infected macrophages. PMA-induced, GFP-, CA-ROCK1- and CA-ROCK2-expressing U937 cells were incubated with atorvastatin or DMSO (vehicle) for 24 h and then incubated with or without *T. cruzi* for 30 min. **(A)** ROCK activation was determined by western blot. **(B, C)** 24 h post-*T. cruzi* incubation, cell media were collected for Multiplex analysis of cytokine secretion, and M1/M2 ratios were determined. **(D–F)** NF-κB activation was determined by western blot as the ratio of phosphorylated (p-p65(Ser536)) to total p65. Data are expressed as mean ± SD of 5 **(B, C)** or 3 **(A, D–F)** independent experiments. *p<0.05, **p<0.01, ***p<0.001, analyzed with two-way ANOVA and Tukey post-test.

In contrast to *T. cruzi*-infected U937-GFP cells, atorvastatin was unable to decrease ROCK activity in *T. cruzi*-infected CA-ROCK1 and CA-ROCK2 U937 macrophages (**Figure 4A**). Atorvastatin decreased the expression and secretion of all M1 markers in GFP-expressing *T. cruzi*-infected U937 cells (**Supplementary Figures 7A, C**). By contrast, it failed to reduce them in CA-ROCK1- or CA-ROCK2-expressing U937 cells infected with *T. cruzi*, except for IL-6 mRNA levels in CA-ROCK2 cells. Moreover, atorvastatin did not change the M2 profile in the GFP, CA-ROCK1 or CA-ROCK2 U937 cells, except for TGF-β mRNA levels, which was augmented by atorvastatin in GFP cells, but not in CA-ROCK1 and CA-ROCK2 U937 cells (**Supplementary Figures 7B, D**). Using M1/IL-10 and M1/IL-4 ratios, atorvastatin promoted an anti-inflammatory M2 profile, whereas CA-ROCK1 and CA-ROCK2 expression prevented this effect (**Figures 4B, C**).

As statins have been shown to decrease NF-κB activation in CD (41), we studied if ROCK is involved in this effect. 10 µM atorvastatin reduced NF-κB activation in GFP U937 cells (**Figure 4D**), but this effect did not occur in CA-ROCK1 (**Figure 4E**) and CA-ROCK2 cells (**Figure 4F**). Thus, these results suggest that atorvastatin decreases NF-κB activation, induces an anti-inflammatory M2 profile, and consequently decreases inflammation by decreasing ROCK activity.

### 3.6 Simvastatin decreases ROCK expression and changes the cytokine profile in the cardiac tissue of chronic chagasic mice

**Figure 5:**
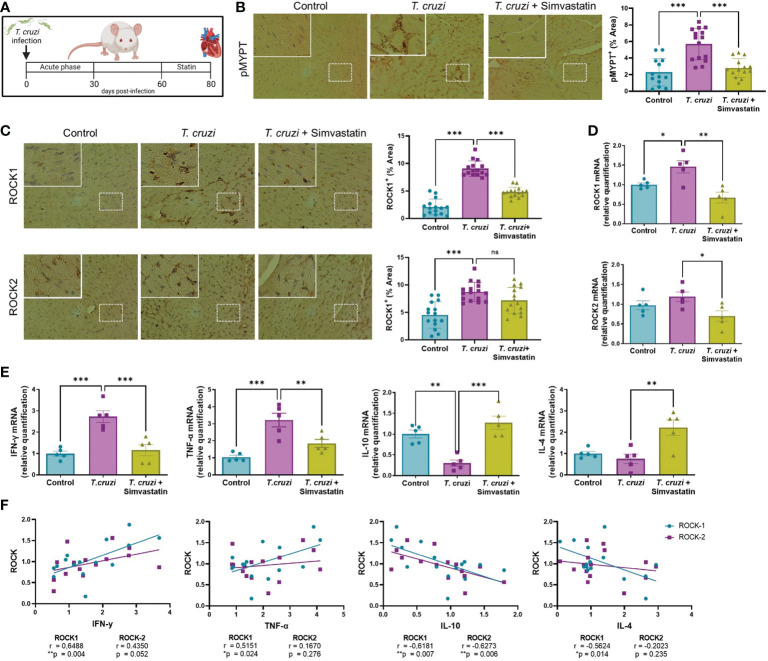
Simvastatin reduces ROCK activation and pro-inflammatory cytokine expression in cardiac tissue of an *in vivo* model of chronic Chagas cardiomyopathy. BALB/c mice were infected with 1 x 10^3^ trypomastigotes of the Dm28 strain. At day 60 post-infection, mice were treated with 1 mg/Kg simvastatin per day for 20 days (25). At day 80 post-infection, mouse hearts were collected for RT-qPCR and immunohistochemistry analysis. **(A)** Experimental murine model. **(B)** Immunohistochemistry for cardiac p-MYPT1 and relative levels. **(C)** immunohistochemistry for cardiac ROCK1 and ROCK2 and relative levels. **(D)** Relative quantification of ROCK1 and ROCK2 mRNA levels. **(E)** Relative quantification of cytokine mRNA levels. Data are expressed as mean ± SD of 5 mice. *p<0.05 **p<0.01 ***p<0.001 analyzed with one-way ANOVA and Dunnett post-test. **(F)** Linear correlation between ROCK and cytokine mRNA levels. *p<0.05, **p<0.01, analyzed with Pearson correlation coefficient (r).

BALB/c mice were treated with 1 mg/Kg/day simvastatin for 20 days, and at day 80th post-infection and heart tissue was evaluated for ROCK and cytokine expression to evaluate the effect of statin treatment on responses to *T. cruzi in vivo* (**Figure 5A**). In this model, simvastatin does not modify cardiac parasite load, although it provided a significant decrease in tissue inflammation (25).

As can be appreciated in **Figure 5B**, the ROCK activity increased in chronic chagasic mice and was decreased by simvastatin. The expression of ROCK proteins in cardiac tissue was also increased in infected mice, although simvastatin decreased only ROCK1 (**Figure 5C**); similarly, ROCK1 but not ROCK2 mRNA levels were increased in the hearts of *T. cruzi*-infected mice (**Figure 5D**). Simvastatin reduced the *T. cruzi*-induced increase in ROCK1 mRNA levels and decreased ROCK2 mRNA levels. This reduction in ROCK1 and ROCK2 transcription after statin treatment was accompanied by a reduction of pro-inflammatory and an increase in anti-inflammatory cytokine expression (**Figure 5E**). A simple linear correlation was carried out to assess the relation between ROCK and relative cytokine mRNA levels. A Pearson correlation coefficient showed that ROCK1 mRNA is positively related to IFN-γ and TNF-α mRNA and negatively related to IL-10 and IL-4 mRNA levels (**Figure 5F**). On the other hand, ROCK2 mRNA levels were only related to IL-10 mRNA levels. These results show that statin treatment in an *in vivo T. cruzi* model decreases ROCK expression and the pro-inflammatory/anti-inflammatory ratio.

## 4 Discussion

The mechanisms underlying the development of CCC are not well known. It has been hypothesized that changes in the immune response due to parasite persistence, inducing impaired cytokine secretion, is one of the determining factors in the progression of the disease to a more severe condition. This study provides the first evidence of the role of ROCKs in CD models. In macrophages, *T. cruzi* rapidly induced ROCK activation (**Figures 1A, B**) and increased ROCK expression at longer times (**Figure 3B**). ROCK expression and activity are increased in leukocytes from rats with hypertension, and there is a correlation between ROCK activity and cardiac hypertrophy and fibrosis (42). ROCK activity is also increased in leukocytes from patients with metabolic syndrome, acute coronary syndrome, diabetes mellitus, and heart failure (43–45), and an inverse relationship between ROCK activation and ejection fraction has been found in patients with heart failure; thus, ROCK activity is a suitable marker to identify patients with a high risk of heart failure (46). Furthermore, we demonstrated that *T. cruzi* infection induced ROCK activation and ROCK1 expression in cardiac tissue of a mouse model of CCC (**Figures 5C, D**), which could contribute to the decrease in heart function as a consequence of the increase in inflammation and fibrosis induced by the parasite (25). An increase in ROCK1 expression is also observed in non-infectious conditions such as ischemia-reperfusion models (47). Moreover, ROCK1 seems to play a role in cardiac fibrosis (48), a major characteristic of chagasic hearts. All these results suggest that ROCK has a role in the development of cardiovascular diseases that could also be occurring in patients with CCC, and therefore ROCK inhibition could prevent and reduce CCC severity.

The mechanism by which *T. cruzi* modulates ROCK activity or expression is unknown; however, it could be mediated by several signaling pathways. It has been reported that LPS activates the RhoA/ROCK pathway by binding to TLR4 (49), a receptor also activated by *T. cruzi*. Our results suggest that *T. cruzi*-induced ROCK activation occurs *via* TLR4 pathway (**Figure 1F**), although other TLRs may be involved. TLR4 is the most studied pattern recognition receptor in *T. cruzi* infection models, and this receptor has been implicated in modulating M1 cytokine levels (50), in agreement with the results presented in our work. The activation of ROCK induced by *T. cruzi* infection was simultaneous with that of NF-κB (41). Moreover, TLR4 inhibition with TAK-242 and ROCK inhibition by Y-27632 prevented the phosphorylation (**Figure 1G**) and nuclear localization of p65 (**Figure 1H**). This suggests that TLR4 acts *via* ROCK to induce NF-κB activation, in agreement with previous reports (51–53). However, TLR4 is not the exclusive innate receptor for interaction with *T. cruzi*, as other receptors of the TLR-like family (54) are also involved in the pro-inflammatory response in macrophages, although it is not known whether they also act *via* ROCK. Because ROCK1 can directly phosphorylate IKKβ at Ser-177 and Ser-181, inducing NF-κB activation (55), this may be the link between *T. cruzi* and NF-κB in our model. Also, RhoA inhibition partially prevented NF-κB activation (**Figure 1I**). In conjunction with its inhibitory effect on ROCK (**Figure 1E**), these results suggest that RhoA activation is upstream of ROCK and NF-κB activation.

Inflammatory processes mediated by cytokines are decisive in the pathogenesis and progression of CCC (9). Lymphocytes and macrophages from patients with CCC secrete higher levels of pro-inflammatory cytokines than asymptomatic chronic Chagasic patients (11); thus, reducing the pro-inflammatory environment is a promising pharmacological strategy in CCC. Although in *in vivo* models of the acute CD there is an increase in M1 and M2 cytokines (56), *in vitro* models have mainly reported an increase in M1 cytokines (57, 58). Nevertheless, our results evidenced an increase of both M1 and M2 markers induced by *T. cruzi in vitro*, as reported by Mendonça et al. (59). Here, the inhibition of ROCK by Y-27632 decreased the expression and secretion of IL-6, IL-1β, and TNF-α (**Figure 2A**). This anti-inflammatory effect of ROCK inhibition has been observed in various other models, both *in vitro* and *in vivo*. For example, ROCK inhibition decreases inflammation in a rheumatoid arthritis model by suppressing the synthesis of TNF-α and IL-1β but not IL-10 (52). A similar result was observed in peripheral blood mononuclear cells (60) and macrophages from mice administered with LPS. In the latter model, Y-27632 decreased TNF-α and increased TGF-β expression (61). Although Y-27632 inhibits both ROCK1 and ROCK2, and thus it is not possible to distinguish which ROCK is involved in our studies, it is possible that both ROCK1 and ROCK2 participate in *T. cruzi*-induced polarization, as it has been observed with siRNAs specific for ROCK1 and ROCK2 that both of them reduce IL-1β production in macrophages (62).

In our model, ROCK-induced NF-κB activation positively regulated M1 and negatively regulated M2 cytokine expression. It has been reported that TLR4-induced NF-κB activation inhibits the Peroxisome Proliferator-Activated Receptor Gamma (PPARγ) (63). The role of PPARγ has been studied in CCC models, where PPARγ agonists increase the expression of M2 markers, reducing inflammation and cardiac fibrosis (64); moreover, Gien et al. (65) suggested that ROCK inactivates PPARγ (65). Thus, the increase in IL-10 and TGF-β mRNA levels induced by Y-27632, as shown in this work (**Figure 2B**), allows speculating that ROCK negatively regulates PPARγ in *T. cruzi-*infected macrophages, although this effect has not been proved yet.

In *T. cruzi*-infected macrophages, ROCK inhibition decreases the secretion of both M1 and M2 markers. In macrophages, cytokine secretion occurs *via* “constitutive exocytosis”, which is dependent on cytokine transcription (66, 67). Vesicle trafficking is involved in various cellular processes, such as cell motility and polarity, in which different Rho GTPases participate. For example, constitutively active RhoA expression increases vesicular secretion in mast cells, and RhoGDI inhibits this activity (68). However, the role of ROCKs in cytokine exocytosis in macrophages has not been studied. Our results suggest that ROCKs could be involved in the secretion of both M1 and M2 cytokines, as inhibition of these kinases decreased the extracellular levels of cytokines of both phenotypes, which is consistent with studies reporting that the pro-inflammatory (IL-6, TNF-α) and anti-inflammatory (IL-10) cytokines have the same secretion pathway (69). Even though both M1 and M2 markers decreased in ROCK-inhibited macrophages, the decreased M1/M2 ratio induced by Y-27632 reveals a drift towards the M2 profile.

Statins inhibit the synthesis of isoprenoids necessary for post-translational modification of RhoA, inhibiting its ability to activate ROCKs. Our results show that atorvastatin inhibited *T. cruzi*-induced ROCK activation (**Figure 3A**) and expression (**Figure 3B**), similar to the effects of statins in various *in vivo* and *in vitro* inflammatory models, such as atherosclerosis (70), and rats administered with LPS (71). Studies that compared the effects of several statins on ROCK activity concluded that ROCK inhibition by statins is class-specific and that both lipophilicity and potency are determining factors in their ability to inhibit ROCK in leukocytes (72, 73). Also, GGPP prevented the atorvastatin-induced ROCK inhibition and M2 polarization (**Figures 3G, H**). This result highlights the role of isoprenoid synthesis in the effect of atorvastatin, although the effect of RhoA prenylation remains to be demonstrated.

The effect of atorvastatin on the expression of M1 markers (**Figure 3C**) in the presence of *T. cruzi*, was like that of the ROCK inhibitor Y-27632 (**Figure 2A**): both molecules decreased mRNA levels and secretion of pro-inflammatory cytokines. The use of statins for cytokine modulation as a pharmacologic strategy has been previously studied (74, 75). In murine models of CCC, simvastatin inhibits NF-κB activation, inflammation, and fibrosis and improves cardiac function (24, 25, 41). This finding is consistent with the decrease in the expression and secretion of pro-inflammatory cytokines we observed with atorvastatin. Moreover, the effect of atorvastatin was attenuated in macrophages expressing CA-ROCK1 or CA-ROCK2 (**Figure 4A**). This result confirms that ROCK1 and ROCK2 mediate the effect of atorvastatin on the synthesis and release of IL-6, IL-1β, and TNF-α.

Statins inhibit NF-κB in LPS-stimulated human monocytes in a dose-dependent manner (76), an effect also observed in *T. cruzi*-infected endothelial cells (41); and, as our results suggest, is mediated by ROCK activity (**Figures 4D–F**). The inability of atorvastatin to inhibit p65 phosphorylation in CA-ROCK1- or CA-ROCK2-expressing macrophages provides additional evidence that statins act *via* ROCK inhibition to alter M1 cytokine expression.

Interestingly, among the M2 markers, atorvastatin only increased TGF-β mRNA levels in *T. cruzi*-infected macrophages (**Figure 3D**). This effect was also prevented by CA-ROCK1 and CA-ROCK2 (**Figure 4B**). As Y-27632 also increased TGF-β mRNA (**Figure 2B**), it is likely that the effect of atorvastatin on this cytokine also involves ROCKs. Interestingly, atorvastatin promotes M2 polarization in human monocytes through the PPARγ activation (77), suggesting that this transcription factor could be involved in the atorvastatin responses we observe. Our results showed that atorvastatin promotes M2 polarization, defined as a reduction in the M1/M2 ratio (**Figures 3E, F**), an effect also prevented by the expression of both CA-ROCK1 and CA-ROCK2 (**Figures 4B, C**). Similar effects of statins in switching macrophage phenotype from M1 to M2 have been observed in LPS-induced macrophage models (78). Furthermore, simvastatin decreased cardiac inflammation in *T. cruzi*-infected dogs. This improvement was accompanied by reduced serum and cardiac mRNA levels of TNF-α and IFN-γ but IL-10 (79), which is consistent with our *in vivo* results (**Figure 5E**). Moreover, in our murine model of CCC, simvastatin promoted a shift in macrophage phenotype toward M2 by changing the cytokine mRNA generation, decreasing M1 cytokines, and increasing M2 (**Figure 5E**). Importantly, this change in cytokine profile was accompanied by a decrease in ROCK1 and ROCK2 cardiac activation and expression (**Figures 5B, C**).

Along with the observed reduction of ROCK activation and expression in cardiac tissue, simvastatin also reduced heart inflammation and fibrosis, and improved ejection fraction in our model (25), but the importance of ROCK in this effect has not been proven yet in *in vivo* CD models. However, heart mRNA levels of ROCK were similarly reduced in a rat isoproterenol-induced chronic heart failure model when treated with atorvastatin while improving cardiac function (80). The effect of statins on heart failure has been extensively studied (81). By decreasing isoprenoid production, statins prevent post-translational modifications of RhoA, downregulating ROCK activation. That, in turn, induces the activation of the PI3K/Akt/eNOS pathway, restoring endothelial function (82), which is important because, in CCC, endothelial dysfunction contributes to the pathogenesis of cardiomyopathy by increasing leukocyte recruitment and inducing local vasoconstriction, with the consequent focal ischemia commonly observed in this pathology (83). Therefore, by decreasing ROCK activation, statins promote the restoration of endothelial function and ameliorate *T. cruzi*-induced inflammation. However, the *in vitro* results showed that atorvastatin similarly decreased ROCK activity by inhibiting RhoA activity with C3 exoenzyme. Whether the effect on ROCK is related directly to RhoA activity *in vivo* remains to be demonstrated.

A limitation of the present work was using a macrophage line of human origin when the validation was performed in a murine model. Using a human line allowed us to approach an actual pathological situation. However, it is not feasible to perform this type of study without the respective preclinical validations before attempting this therapy in humans. Additionally, the effect of *T. cruzi* on U937 macrophages is closer to an acute infection scenario, whereas the cardiac results were done in a chronic model. Nevertheless, the atorvastatin-induced changes in the cellular model were replicated in the chronic murine model as a proof-of-concept, opening a window of possibility to explain, at least in part, the potential benefit of statin use in the chronic phase of this disease.

In conclusion, ROCK expression and activation participate in the immune response of CD, modulating macrophage phenotype, and promoting a pro-inflammatory response, which may be responsible for the cardiac complications of CCC. Statin-triggered ROCK inhibition prevents the pro-inflammatory environment by inducing an immunoregulatory M2-like polarization. This statin-induced immunoregulation, combined with specific trypanocidal therapy, could be a feasible pharmacological strategy in the treatment of CCC.

## Data availability statement

The original contributions presented in the study are included in the article/**Supplementary Material**. Further inquiries can be directed to the corresponding authors.

## Ethics statement

The animal study was reviewed and approved by Bioethics Committee of the Faculty of Medicine, University of Chile (CBA 0937 FMUCH protocol).

## Author contributions

FG-H, JM, AR, RV contributed to the conception and design of the study. FG-H, NC, DG-R, IC, HQ-P, MR-M, MC and RV performed the experimental approach. FG-H, AR, CC, CO-A, UK, RV, JM contributed to the first draft of the manuscript. MC, RA contributed to the statistical analysis. All authors contributed to the article and approved the submitted version.
