# Supplementary material for: Statins change the cytokine profile in Trypanosoma cruzi-infected U937 macrophages and murine cardiac tissue through Rho-associated kinases inhibition
Source: Front Immunol. 2023 Jan 11;13:1035589. doi: 10.3389/fimmu.2022.1035589 (PMC9874148; doi:10.3389/fimmu.2022.1035589)
Supplement: Supplementary file 1 [file DataSheet_1.docx]

Supplementary Figures


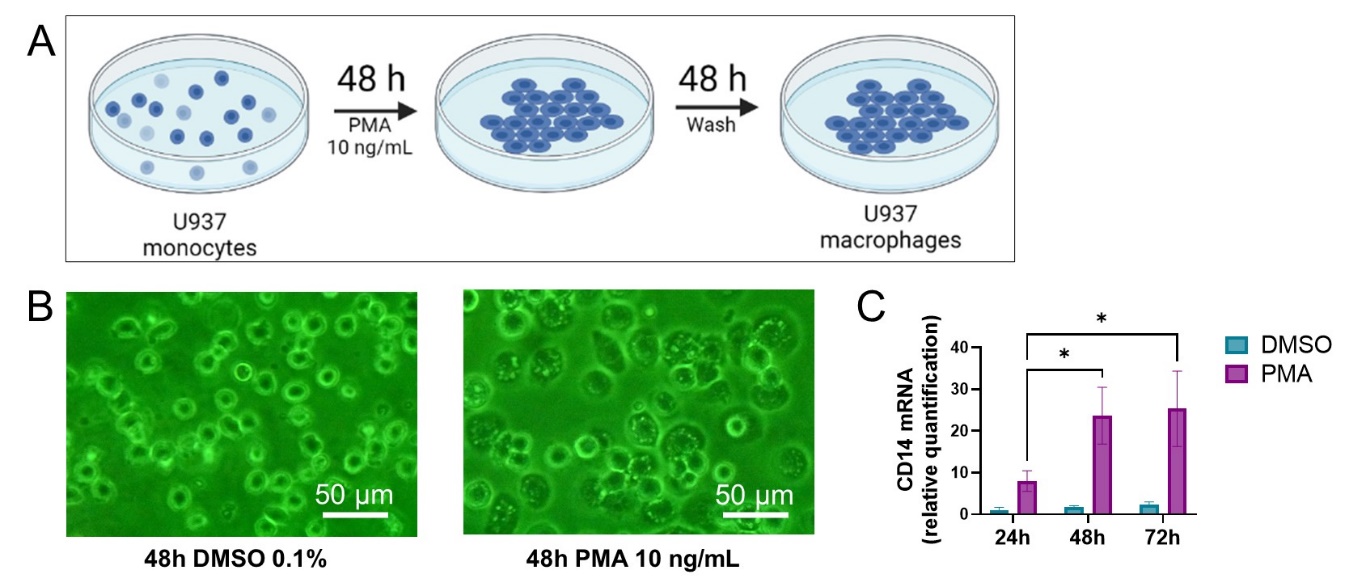


**Supplementary figure 1. PMA-induced U937 differentiation.** **(A)** Before each experiment, U937 cells were incubated with PMA (10 ng/mL) for 48 h, then washed and changed for new cell media for 48 h. **(B)** U937 cells were incubated with PMA (10 ng/mL) or vehicle (DMSO 0.1%) for 48 h. Adherent cells show differentiated macrophages. **(C)** U937 cells were incubated with PMA (10 ng/mL) or vehicle (0.1% DMSO) for 24, 48 or 72 h. Differentiation to macrophages was determined with RT-qPCR analysis evaluating CD14 mRNA levels. Data are expressed as mean ± SD of 3 independent experiments. *p<0.05, analyzed by two-way ANOVA and Tukey post-test.


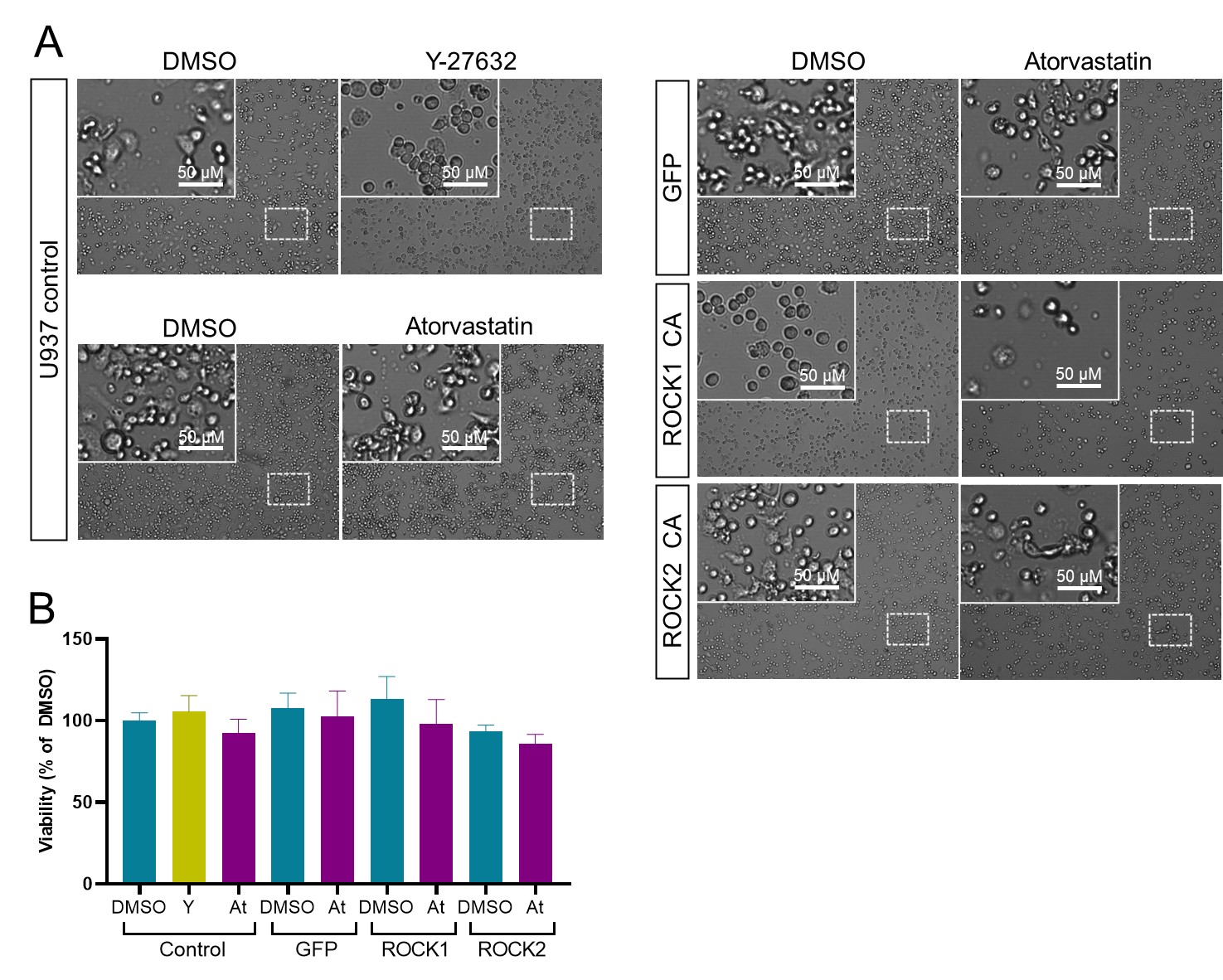


**Supplementary figure 2. Morphology and viability of atorvastatin and Y-27632-incubated U937 cells. (A)** PMA-induced U937 cells were incubated with the ROCK inhibitor Y-27632, atorvastatin or DMSO for 24 h. **(B)** U937 cell viability was measured by MTT, relative to Control DMSO. Data are expressed as mean ± SD of 3 independent experiments. Analyzed with one-way ANOVA and Tukey post-test.


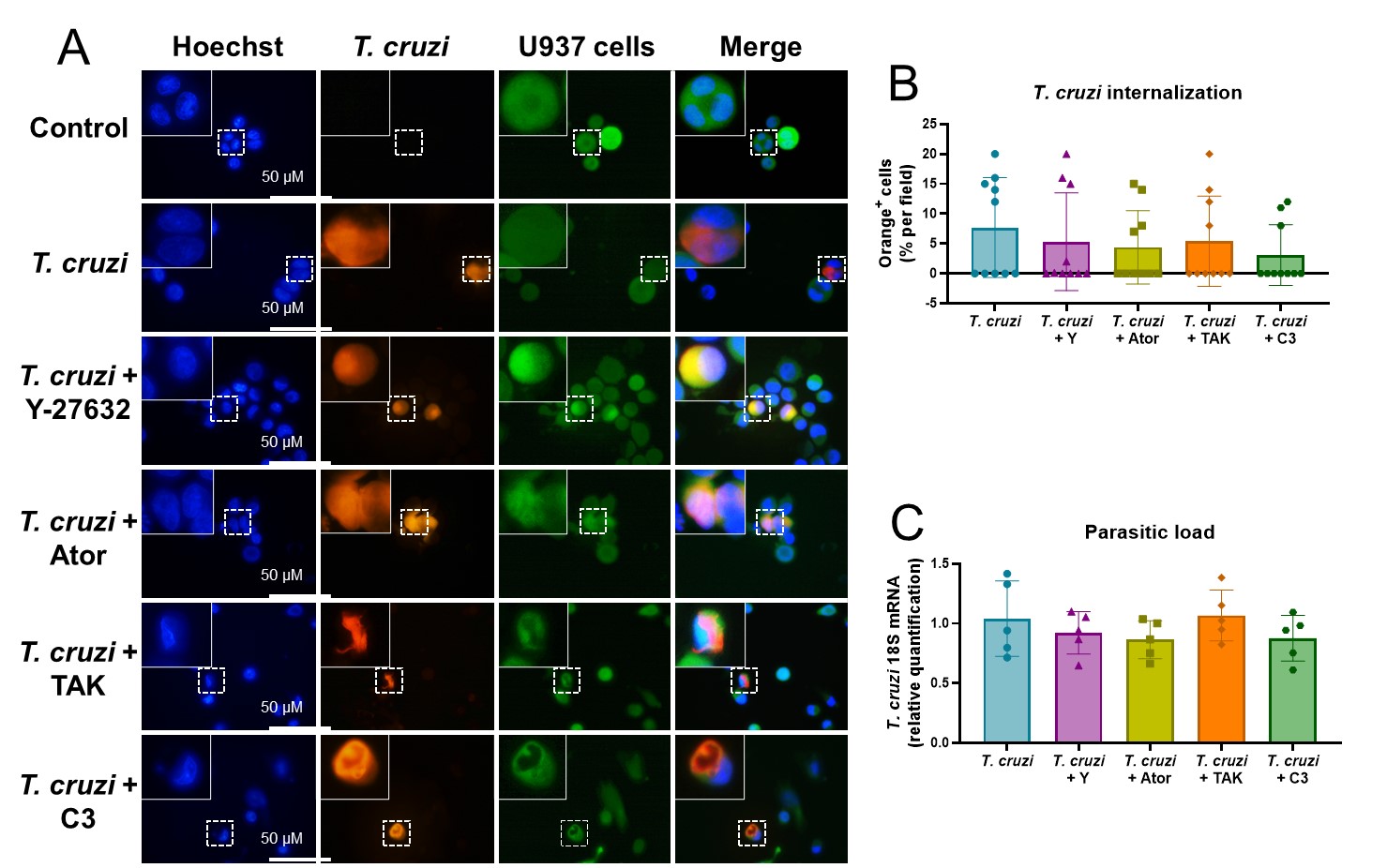


**Supplementary figure 3. *T. cruzi* internalization in treated U937 macrophages. (A)** CellTracker green-labeled PMA-induced U937 cells were incubated with Y-27632, atorvastatin (Ator), TAK-242 (TAK), or C3 exoenzyme (C3) and subsequently, incubated with CellTracker orange-labbelled *T. cruzi* for 30 min. Non-infected cells were used as control. Then, cells were washed with PBS, and a fresh medium was added. 24 hours post-infection, cells were fixed and labeled with Hoechst for epifluorescence microscope photography. **(B)** Percentaje of CellTracker orange possitive cells per field was measured in 10 field per condition. **(C)** Parasitic load was measured by *T. cruzi* 18S RNA levels determinated by RT-qPCR, relative to human GAPDH. Data are expressed as mean ± SD of 5 independent experiments.


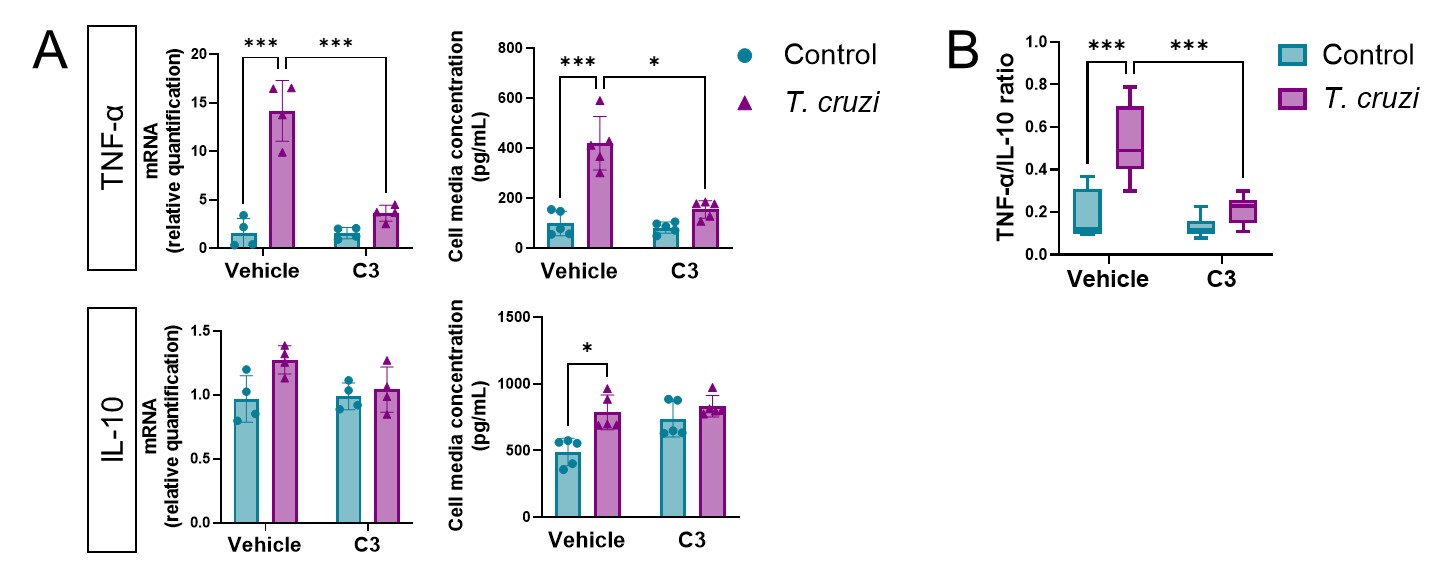


**Supplementary figure 4. RhoA inhibition reduces M1 polarization in T. cruzi-infected macrophages.** **(A)** PMA-induced U937 cells were incubated with C3 exoenzyme (C3) or vehicle (C3 buffer) for 24 h, and then incubated with or without T. cruzi for 30 min. 6 h post-infection, total RNA was extracted for RT-qPCR analysis, and 24 h post-infection, cell media were collected for ELISA analysis. **(B)** Secreted TNF-α to IL-10 ratio. Data are expressed as mean ± SD of 5 independent experiments. *p<0.05, analyzed by two-way ANOVA and Tukey post-test.


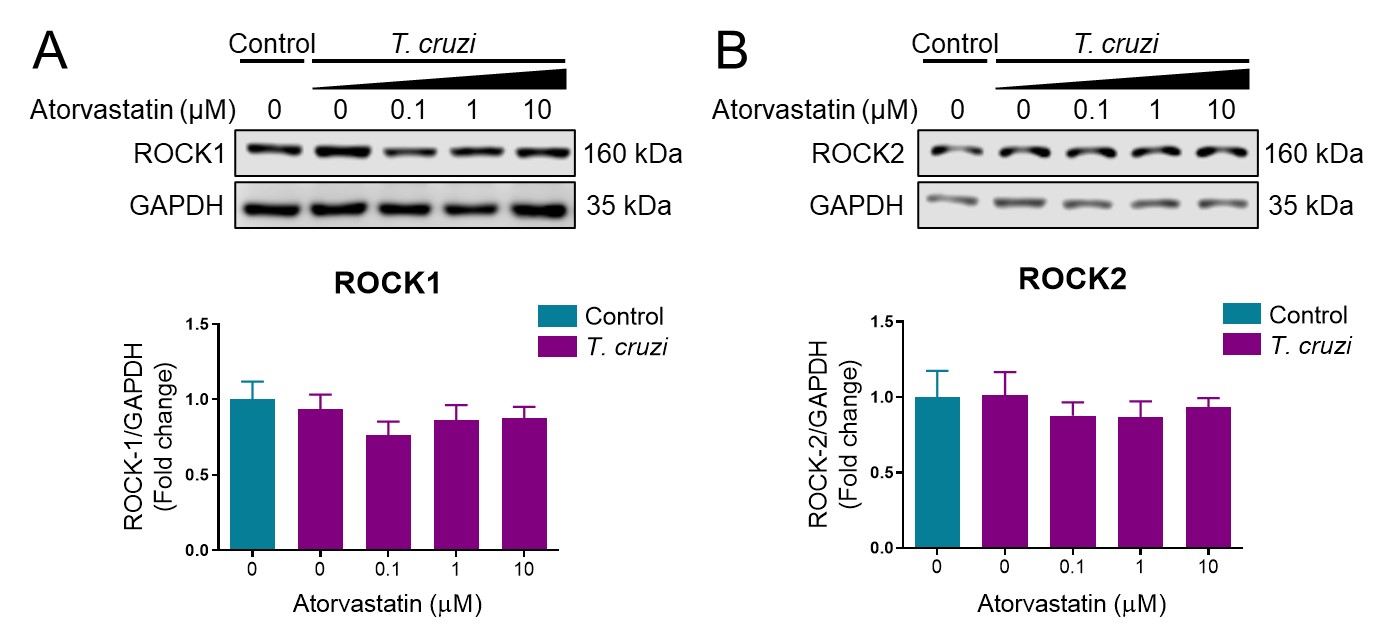


**Supplementary figure 5. Atorvastatin does not change ROCK1 and ROCK2 protein levels at short times of *T. cruzi* infection.** PMA-induced U937 cells were incubated with atorvastatin at increasing concentrations during 24 h and subsequently incubated with or without *T. cruzi* for 30 min. ROCK1 **(A)** and ROCK2 **(B)** protein levels were assessed by western blot. Data are expressed as mean ± SD of 3 independent experiments.


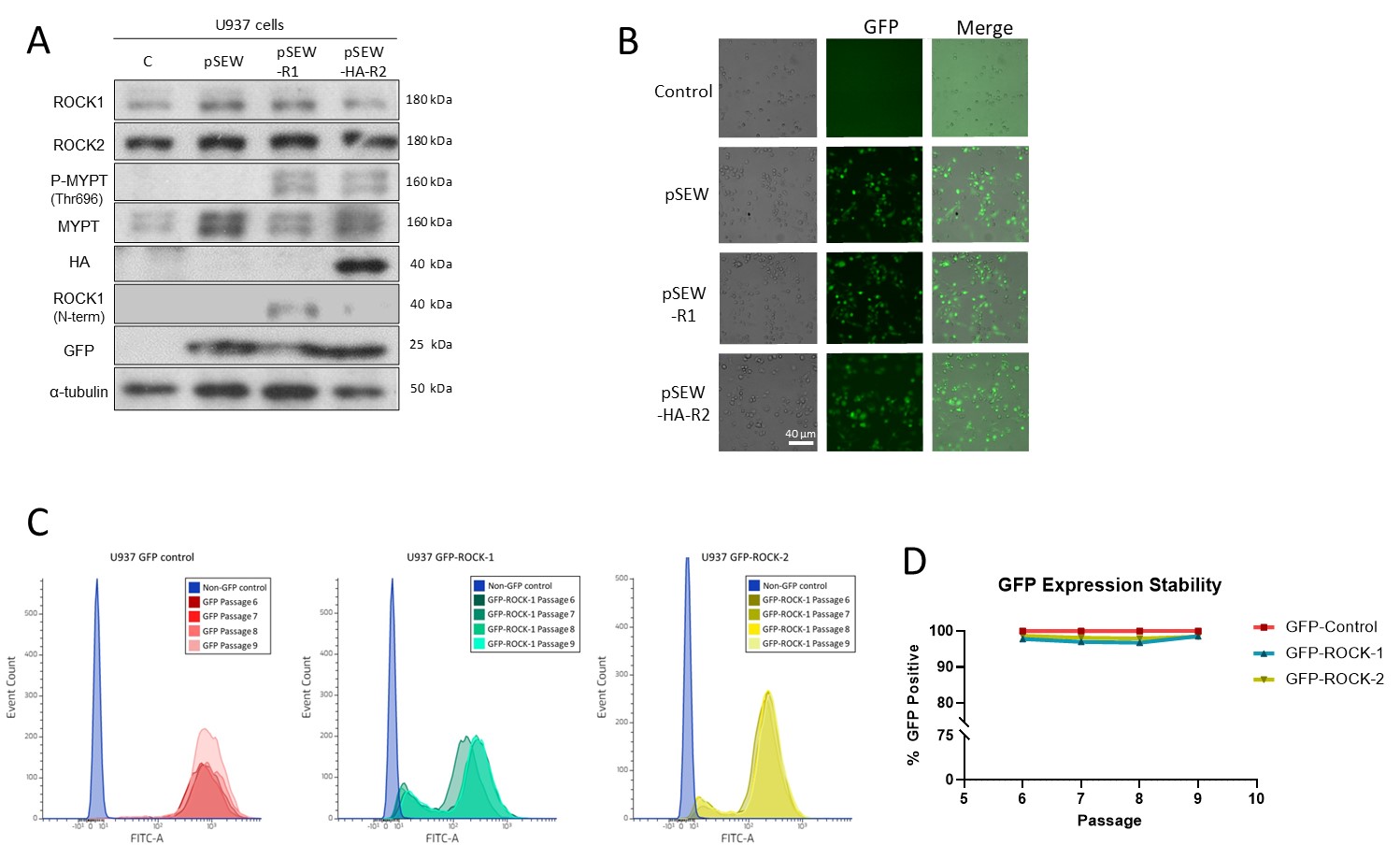


**Supplementary figure 6. Constitutively active-expressing U937 cells model validation.** **(A)** After lentiviral-induced expression of GFP, GFP-CA ROCK1 and GFP-CA ROCK2 in U937 cells, cells were lysed to determine protein levels by western blot: WT ROCK1 and ROCK2 (180 kDa), as well as p-MYPT1 and MYPT1 (160 kDa). CA-ROCK1 levels were determined by an anti-N-terminal ROCK1 antibody (40 kDa), CA-ROCK2 was determined with an anti-HA antibody (40 kDa) and GFP expression was determined with an anti-GFP antibody (25 kDa), as well as epifluorescence microscopy **(B)**. **(C)** Stability of GFP expression during passages was determined by FACS (FITC). Histograms shows FITC^+^ cells of non-GFP-transduced control U937 cells, and lentivirus-induced GFP expression during passage 6 to 9. **(D)** Graphic shows % of FITC^+^ cells per passage.


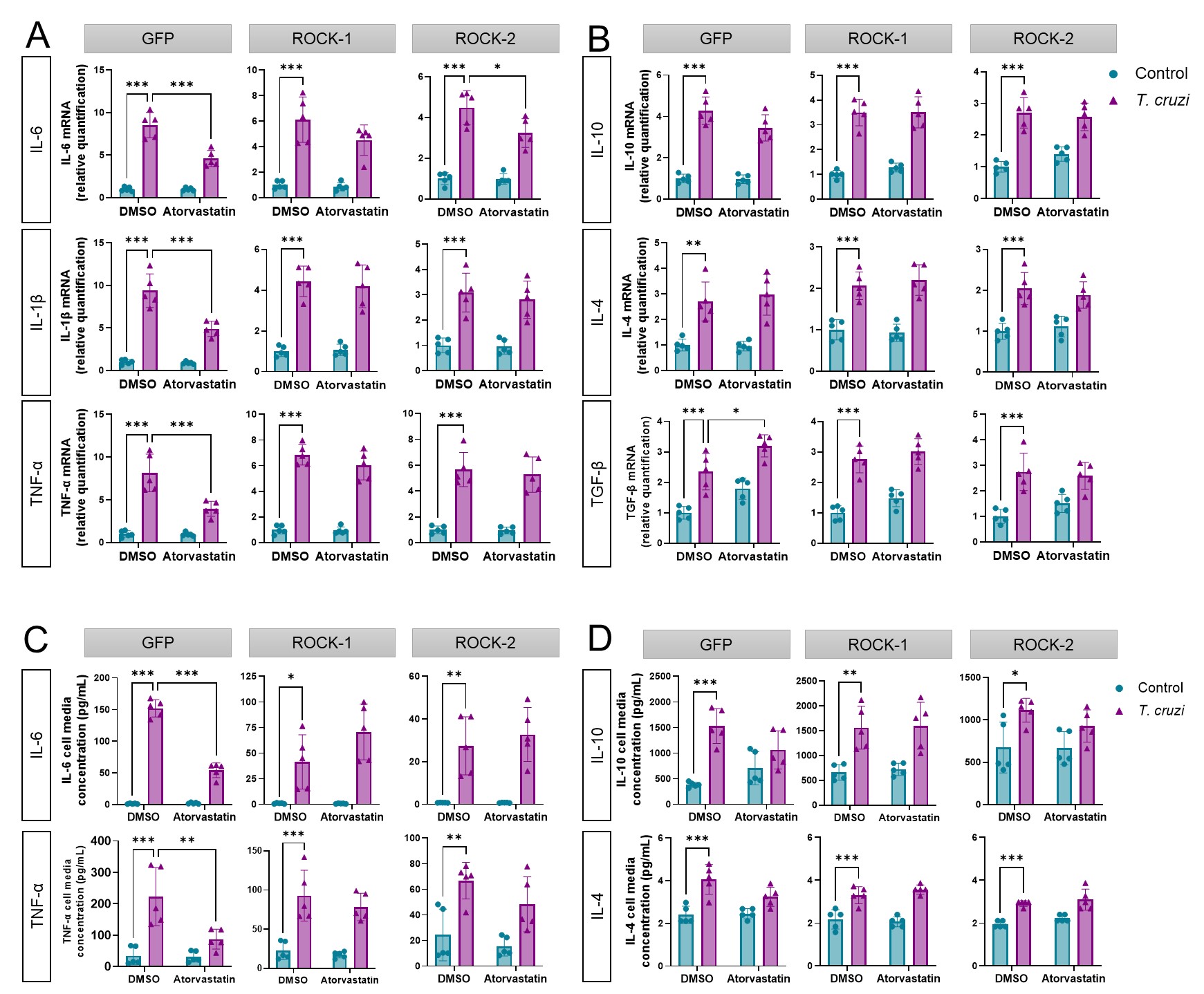


**Supplementary figure 7. CA expression of ROCK-1 and ROCK-2 U937 prevents the antiinflammatory effect induced by atorvastatin.** PMA-induced, GFP, CA ROCK-1 and CA ROCK-2 expressing U937 cells were incubated with atorvastatin or DMSO (vehicle) for 24 hours, and then incubated with or without *T. cruzi* for 30 min. After 6 hours post- *T. cruzi* incubation, RNA was extracted for RT-qPCR analysis of M1 **(A)** and M2 **(B)** cytokines. After 24 hours post-*T. cruzi* incubation, cell media was collected for Multiplex analysis of M1 **(C)** and M2 **(D)** cytokines. Data are expressed as mean ± SD of 5 independent experiments. *p<0.05 **p<0.01 ***p<0.001 analyzed with two-way ANOVA and Tukey post-test.
